# Development and Validation of a Nomogram Based on Nutritional Indicators and Tumor Markers for Prognosis Prediction of Pancreatic Ductal Adenocarcinoma

**DOI:** 10.3389/fonc.2021.682969

**Published:** 2021-05-31

**Authors:** Haoran Li, Fang Zhou, Zhifei Cao, Yuchen Tang, Yujie Huang, Ye Li, Bin Yi, Jian Yang, Peng Du, Dongming Zhu, Jian Zhou

**Affiliations:** ^1^ Department of General Surgery, The First Affiliated Hospital of Soochow University, Suzhou, China; ^2^ Department of General Surgery, Changshu No. 2 People’s Hospital, Suzhou, China; ^3^ Department of Pathology, The Second Affiliated Hospital of Soochow University, Suzhou, China; ^4^ Department of General Surgery, The Second Affiliated Hospital of Soochow University, Suzhou, China

**Keywords:** nomogram, CONUT, PNI, pancreatic ductal adenocarcinoma, prognosis

## Abstract

**Purpose:**

This study aimed to develop and validate a nomogram with preoperative nutritional indicators and tumor markers for predicting prognosis of patients with pancreatic ductal adenocarcinoma (PDAC).

**Methods:**

We performed a bicentric, retrospective study including 155 eligible patients with PDAC. Patients were divided into a training group (n = 95), an internal validation group (n = 34), an external validation group (n = 26), and an entire validation group (n = 60). Cox regression analysis was conducted in the training group to identify independent prognostic factors to construct a nomogram for overall survival (OS) prediction. The performance of the nomogram was assessed in validation groups and through comparison with controlling nutritional status (CONUT) and prognostic nutrition index (PNI).

**Results:**

The least absolute shrinkage and selection operator (LASSO) regression, univariate and multivariate Cox regression analysis revealed that serum albumin and lymphocyte count were independent protective factors while CA19-9 and diabetes were independent risk factors. The concordance index (C-index) of the nomogram in the training, internal validation, external validation and entire validation groups were 0.777, 0.769, 0.759 and 0.774 respectively. The areas under curve (AUC) of the nomogram in each group were 0.861, 0.845, 0.773, and 0.814. C-index and AUC of the nomogram were better than those of CONUT and PNI in the training and validation groups. The net reclassification index (NRI), integrated discrimination improvement (IDI) and decision curve analysis showed improvement of accuracy of the nomogram in predicting OS and better net benefit in guiding clinical decisions in comparison with CONUT and PNI.

**Conclusions:**

The nomogram incorporating four preoperative nutritional and tumor markers including serum albumin concentration, lymphocyte count, CA19-9 and diabetes mellitus could predict the prognosis more accurately than CONUT and PNI and may serve as a clinical decision support tool to determine what treatment options to choose.

## Introduction

Pancreatic ductal adenocarcinoma (PDAC) is one of the most intractable malignant neoplasms worldwide (1). Surgical resection is the only potentially curative option for stage I/II PDAC; nonetheless, recurrence would happen in most patients after surgery (2), indicating that it is necessary to identify the factors affecting postoperative prognosis in patients with PDAC. In recent years, many clinical factors are found to be associated with the prognosis of PDAC, such as tumor markers, cancer stage and chemotherapy (3, 4).

In addition to tumor markers and cancer stage, preoperative factors including inflammation and nutritional status are identified to have effect on survival after surgery (5). Nutrition has become one of the most popular indices to assess the prognosis of patients with cancer. Lee et al. (6) reported that there is a correlation between prognostic nutrition index (PNI) and the survival time of patients with pancreatic cancer. Kato et al. (7) also found that the controlling nutritional status (CONUT) existed as an independent risk factor for survival in patients with pancreatic cancer. However, both studies were not initially and specifically established for PDAC and they didn’t include specific clinical factors for PDAC, restricting themselves in prediction of prognosis in patients with PDAC. In clinic, more comprehensive and accurate nutritional screening and assessment tools such as subjective global assessment and patient-generated subjective global assessment, require the intervention of trained professionals and are time-consuming, making them difficult to be widely used within inpatients and outpatients. Considering this, developing a convenient and specific model for predicting prognosis of patients with PDAC is of great necessity.

This study aimed to identify nutritional indicators and tumor markers and establish a specific and convenient nomogram to predict 2-year overall survival (OS) of patients with PDAC after surgical resection and to compare the accuracy of the nomogram with that of CONUT and PNI. Our target was to provide surgeons and patients with precise prediction of survival and facilitate clinical decision-making.

## Materials And Methods

### Patients

This retrospective study enrolled 129 stages I–III pancreatic ductal adenocarcinoma patients who underwent surgical resection at the Department of General Surgery, the First Affiliated Hospital of Soochow University, between April 2012 and July 2017, while excluded the patients who had received preoperative chemotherapy, transfusion, or suffered from immune diseases. The patients lost to follow up were also excluded. The median age of these 129 patients was 65 (range 36–80). We collected clinical data of 95 patients from April 2012 to December 2016 as the training group and 34 patients from January 2017 to July 2017 as the internal validation group from the First Affiliated Hospital of Soochow University. According to the same standard, we collected 26 cases from January 2017 to December 2017 as the external validation group from the Second Affiliated Hospital of Soochow University. In the external validation group, the median age of patients was 67.5 (range 45–82). Besides, the entire validation group was incorporated by internal validation group and external validation group. In total, this study enrolled 155 patients, of whom resected pancreatic tumors were pathologically confirmed as ductal adenocarcinoma. This study was conducted in accordance with the Declaration of Helsinki and was approved by the ethics committee of the First Affiliated Hospital of Soochow University. Informed consent was obtained from all the patients.

### Treatment

In our surgery center, diagnostic imaging exams were performed before surgery for avoiding surgery in patients with distant metastasis and incurable resection. Surgical methods were pancreaticoduodenectomy and distal pancreatectomy and resected tissues were pathologically examined in frozen sections to confirm diagnosis and negative surgical margins.

Adjuvant chemotherapy including gemcitabine plus capecitabine or gemcitabine plus S-1 (tegafur, gimeracil, and oteracil potassium) was started within 3 months after the surgery if necessary.

### Follow Up

OS was defined as the time from surgery to death in any case. Postoperative follow-up assessments were performed every 3 months for 2 years and OS was collected until December 31, 2019. The median follow-up periods of patients from the First Affiliated Hospital of Soochow University and the Second Affiliated Hospital of Soochow University were 18 (range 3.0–54.2) months and 17.5 (range 3.0–53.0) months, respectively. Causes of death and clinical data were determined by reviewing medical records, including laboratory data, CT/MRI results and telephone follow-up. The follow-up rate of our study was 96.27%.

### Investigational Variables

We collected data on preoperative blood tests within three days before surgery, including biochemical examination and blood routine examination. The following demographic data were analyzed: gender, age, body mass index (BMI), tumor diameter, lymphocyte, albumin, hemoglobin, platelet, total bilirubin, aspartate transaminase, alanine transaminase, pre-albumin, C-reactive protein (CRP), carbohydrate antigen 19-9 (CA19-9), surgical methods, tumor location, tumor-node-metastasis (TNM) stage, pathological differentiation, 2-year survival, median survival time, diabetes and chemotherapy.

CONUT score was calculated based on serum albumin, total cholesterol and lymphocyte count. The patients were divided into three different groups according to CONUT score: normal (0–1 scores), light (2–4 scores), moderate (5–8 scores) and severe (9–12 scores) (8).

PNI score was calculated using serum albumin and lymphocyte count. The formula was PNI = albumin (g/L) + 5 × lymphocyte (10^9^/L) (9). The patients were divided into two groups according to the best cut-off value of the PNI scores using the receiver operating characteristic (ROC) curves.

### Statistical Analysis

Statistical comparisons between groups were performed with analysis of Variance or Mann–Whitney U test for continuous variables and the Chi-square test or Fisher’s exact test for categorical variables. The Kaplan–Meier was applied to construct OS curves which was then compared by log-rank test. Factors with *p <*0.1 were selected into the multivariate Cox proportional hazards model, of which hazard ratios (HRs) and 95% confidence intervals (CIs) were calculated. The least absolute shrinkage and selection operator (LASSO) analysis was used to select potential factors for prediction of prognosis. We used factors selected from the training group to construct a nomogram using rms R package. In order to value the discrimination ability, the nomogram was subjected to bootstrapping validation (1,000 bootstrap resamples) to calculate a relatively corrected concordance index (C-index). Subsequently, we used calibration curve to assess the calibration of the nomogram. Time-dependent ROC, net reclassification index (NRI) and integrated discrimination improvement (IDI) were applied to compare predictive accuracy of the nomogram with PNI and CONUT in the training group. The same comparisons were then performed in the internal validation, external validation and external validation groups.

Both NRI and IDI are novel techniques to assess the incremental improvement in prediction over a baseline prediction model (10, 11). NRI, proposed by Pencina et al. in 2008 (10), was conducted to evaluate the improvement of predictive accuracy of our model compared with CONUT and PNI. IDI, which evaluates how much an individual’s predicted probabilities changes with the applying of different models (12), was performed to assess the improvement of our model in sensitivity without sacrifice of specificity as compared with CONUT and PNI. The decision curve analysis was applied to quantify the clinical usefulness of the nomogram in the training, internal validation, external validation and entire validation groups.

All statistical analysis was performed by SPSS 22 for Windows (IBM, Armonk, NY, United States) and R software (version 3.5.1; http://www.Rproject.org). The level of statistical significance was set at *p <*0.05.

## Results

### Clinical Characteristics

The demographic and clinical characteristics of 155 eligible patients with pancreatic ductal adenocarcinoma underwent radical resection are shown in **Table 1**, including 95 patients in the training group, 34 patients in the internal validation group and 26 patients in the external validation group. Patients in three groups showed no significant differences in clinicopathological characteristics including gender, age, BMI, tumor diameter, albumin, hemoglobin, platelet, total bilirubin, aspartate transaminase, alanine transaminase, pre-albumin, CRP, CA19-9, surgical methods, differentiation, 2-year survival, median survival time and chemotherapy, whereas different in lymphocyte, tumor location, and diabetes. The average of lymphocyte was 1.36 × 10^9^/L for 95 patients in the training group, *versus* 1.34 × 10^9^/L and 1.20 × 10^9^/L in the internal and external validation groups, respectively. Among the external validation group, patients with tumors located in pancreatic body are the least (n = 2, 7.69%), compared with patients with tumors in pancreatic head and tail. However, in the training and internal validation groups, patients with tumors located in pancreatic tail are the least. Additionally, less patients in the external validation group than training group and internal validation group were free of diabetes. Detailed patient characteristics are given in **Table 1**.

**Table 1:** Demographic and clinical characteristics of patients in the training and validation groups.

	Training (n = 95)	Internal (n = 34)	External (n = 26)	*p*
**Gender, (%)**				0.468
Male	54 (56.84%)	23 (67.65%)	17 (65.38%)	
Female	41(43.16%)	11 (32.35%)	9 (34.62%)	
**Age, years**	64.12 ± 9.56	67.65 ± 8.12	65.30 ± 9.73	0.166
**BMI, kg/m^2^**	21.29 ± 2.24	21.73 ± 1.54	21.23 ± 1.58	0.558
**Tumor diameter, cm**	3.89 ± 1.81	3.25 ± 1.44	3.35 ± 1.28	0.302
**Lymphocyte, 10^9^/L**	1.36 ± 0.58	1.34 ± 0.53	1.20 ± 0.53	**<0.001**
**Albumin, g/L**	38.05 ± 5.57	38.24 ± 5.69	38.09 ± 4.14	0.963
**HB, g/L**	125.75 ± 13.90	126.18 ± 18.44	126.89 ± 16.76	0.948
**PLT, 10^9^/L**	215.93 ± 70.23	197.85 ± 62.91	190.19 ± 53.00	0.136
**T-BIL, μmol/L**	39.70	22.00	64.70	0.558
**AST, μmol/L**	89.39 ± 92.11	96.89 ± 86.60	89.15 ± 110.75	0.919
**ALT, μmol/L**	132.4 ± 162.91	129.82 ± 122.19	113.50 ± 133.96	0.850
**Pre-Alb, mg/L**	153.29 ± 56.35	165.12 ± 38.54	164.00 ± 64.55	0.118
**CRP, mg/L**	4.87 ± 4.27	5.24 ± 4.17	5.49 ± 3.62	0.659
**CA19-9, U/ml**	370.77 ± 361.78	316.91 ± 398.95	388.49 ± 454.91	0.706
**Surgical methods, (%)**				0.198
PD	68 (71.58%)	28 (82.35%)	16 (61.54%)	
DP	27 (28.42%)	6 (17.65%)	10 (38.46%)	
**Tumor location, (%)**				**0.018**
Head	58 (61.05%)	27 (79.41%)	15 (57.69%)	
Body	22 (23.16%)	5 (14.71%)	2 (7.69%)	
Tail	15 (15.79%)	2 (5.88%)	9 (34.62%)	
**TNM stage, (%)**				0.451
I	31 (32.63%)	9 (26.47%)	11 (42.31%)	
II	50 (52.63%)	16 (47.06%)	11 (42.31%)	
III	14 (14.74%)	9 (26.47%)	4 (15.38%)	
**Pathological differentiation, (%)**				0.521
High/Medium	55 (57.89%)	19 (55.88%)	18 (69.23%)	
Low	40 (42.11%)	15 (44.12%)	8 (30.77%)	
**2-year survival, (%)**				0.565
Yes	30 (31.58%)	14 (41.18%)	8 (30.77%)	
No	65 (68.42%)	20 (59.82%)	18 (69.23%)	
**Median survival time, month**	16.6	20	17.5	0.380
**Diabetes, (%)**				**<0.001**
Yes	19 (20.00%)	7 (20.60%)	15 (57.69%)	
No	76 (80.00%)	27 (79.40%)	11 (42.31%)	
**Chemotherapy (%)**				0.440
Yes	30 (31.58%)	14 (41.18%)	11 (42.31%)	
No	65 (68.42%)	20 (58.82%)	15 (57.69%)	

BMI, body mass index; HB, hemoglobin; PLT, platelet; T-BIL, total bilirubin; AST, aspartate transaminase; ALT, alanine transaminase; Pre-Alb, pre-albumin; CRP, C-reactive protein; CA, carbohydrate antigen; TNM, tumor-node-metastasis staging. Bold represents P-value of the variable is less than 0.05.

### Nutrition Status

The 95 patients in training group were divided into three different groups according to the criteria of the CONUT score. There were 26, 47 and 22 patients in normal (0–1 scores), light (2–4 scores) and moderate (5–8 scores) and severe groups (9–12 scores), respectively.

The 2-year survival rates of different CONUT groups were 65.4, 25.5 and 4.6%, respectively (**Figure S1A**). The median OS of the normal, light and moderate and severe groups were 34.8, 16.5 and 9.2 months (**Figure S1B**, *p <*0.001). According to PNI score, the 2-year survival rates were 52.9 and 6.8% in the high and low PNI groups, respectively (**Figure S1C**), and the median OS of high PNI group and low PNI group were 23.8 and 11.7 months (**Figure S1D**, *p <*0.01).

### Selection of Factors Associated With Prognosis and Construction of a Nomogram for PDAC

**Figure 1:**
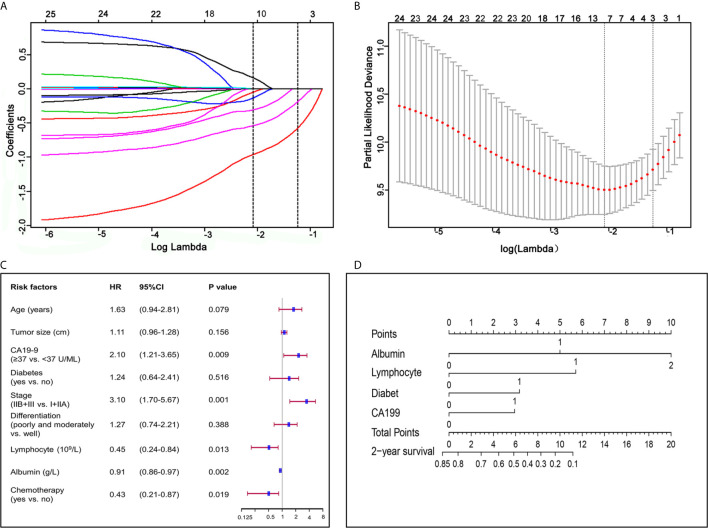
Factors associated with prognosis and construction of a nomogram for pancreatic ductal adenocarcinoma. **(A)** LASSO coefficient profiles of the fractions of 17 preoperative indicators and clinicopathological factors associated with pancreatic ductal adenocarcinoma. **(B)** Tenfold cross-validation for tuning parameter selection in the LASSO model. **(C)** Forest plot visualizing the HRs of prognostic factors identified by multivariate COX analysis of the training group. **(D)** Nomogram for predicting 2-year survival for patients in the training group. The nomogram was established in the training group, with albumin, lymphocyte, diabetes and CA19-9 incorporated.

LASSO was undertaken to identify independent prognosis-related factors. Gender, age, BMI, tumor diameter, stage, differentiation, surgical methods, chemotherapy, lymphocyte count, albumin, CA19-9, hemoglobin, platelet, aspartate transaminase, alanine transaminase, pre-albumin, and CRP in the training group were included in LASSO regression. With lambda of 0.125, 6 key factors were identified to be of great significance to the prognosis of patients with pancreatic ductal adenocarcinoma, including chemotherapy, TNM stage, diabetes, albumin, lymphocyte count and CA19-9 (**Figures 1A, B**).

Our univariate COX regression analysis revealed that age, BMI, tumor diameter, lymphocyte count, albumin, CA19-9, diabetes, TNM stage, differentiation, and chemotherapy were associated with prognosis of patients (**Table 2**). In multivariate COX regression analysis of all variables, independent prognosis-related factors were lymphocyte count (HR = 0.449; *p* = 0.013), albumin (HR = 0.912; *p* = 0.002), CA19-9 (HR = 2.100; *p* = 0.009), TNM stage (HR = 3.102; *p <*0.001) and chemotherapy (HR = 0.430; *p* = 0.019), among which lymphocyte count, albumin and chemotherapy were protective factors, while CA19-9 and TNM stage were risk factors (**Table 2** and **Figure 1C**).

**Table 2:** Univariate and multivariate analyses of predictive factors for 2-year overall survival in the training group (n = 95).

	Univariate analysis				Multivariate analysis		
	COEF	HR	P-value		COEF	HR	P-value
**Gender (%)**							
Female		1 (reference)					
Male	0.2380	1.268	0.352				
**Age, year**					0.489	1.630	0.079
<65		1 (reference)					
≥65	0.4720	1.604	0.059				
**BMI, kg/m^2^**	−0.133	0.876	**0.031**		−0.052	0.949	0.454
**Tumor diameter, cm**	0.1450	1.156	0.013		0.105	1.110	0.156
**Lymphocyte,10^9^/L**	−1.2660	0.282	<0.001		−0.801	0.449	**0.013**
**Albumin, g/L**	−0.1360	0.873	<0.001		−0.093	0.912	**0.002**
**HB, g/L**	−0.0060	0.994	0.472				
**PLT,10^9^/L**	−0.0030	0.997	0.16				
**AST, μmol/L**	−0.0022	1.000	0.868				
**ALT, μmol/L**	0.0002	1.000	0.985				
**Pre-Alb, mg/L**	−0.0020	0.998	0.453				
**CRP, mg/L**	0.0030	1.003	0.91				
**CA19-9, U/ML**					0.742	2.1	**0.009**
Low		1 (reference)					
High	0.7320	2.079	0.007				
**Diabetes (%)**					0.219	1.244	0.516
No		1 (reference)					
Yes	0.6830	1.979	0.016				
**Tumor location (%)**							
Head		1 (reference)					
Body	0.145	1.156	0.693				
Tail	−0.161	0.851	0.711				
**Surgical methods (%)**							
DP		1 (reference)					
PD	−0.312	0.732	0.290				
**TNM stage (%)**					1.132	3.102	**<0.001**
I + IIA		1 (reference)					
IIB + III	1.027	2.791	0.008				
**Pathological differentiation (%)**					0.242	1.273	0.388
High/moderate		1 (reference)					
Low	0.5190	1.680	0.037				
**Chemotherapy (%)**					−0.844	0.430	**0.019**
No		1 (reference)					
Yes	−0.629	0.533	0.020				

COEF, coefficient; HR, hazard ratio; BMI, body mass index; HB, hemoglobin; PLT, platelet; AST, aspartate transaminase; ALT, alanine transaminase; Pre-Alb, pre-albumin; CRP, C-reactive protein; CA, carbohydrate antigen; TNM, tumor-node-metastasis staging. Bold represents P-value of the variable is less than 0.05.

Considering that we aimed to establish a convenient and specific model for predicting OS of patients with resected PDAC, four accessible preoperative factors including albumin, lymphocyte count, diabetes and CA19-9 were selected and incorporated to establish a nomogram for predicting 2-year OS of patients with resected PDAC (**Figure 1D**). Meanwhile, we conducted Kaplan–Meier survival analysis to these four preoperative factors, and the survival curve showed that patients with lower serum albumin (≤35 g/L), higher CA19-9 (≥37 U/ml), lower lymphocyte count (<1.5 × 10^9^/L), or diabetes had poorer prognosis and shorter median survival time (**Figure S2**).

### ROC Curve and C-Index in Training and Validation Groups

To evaluate and validate the predictive ability of our novel nomogram, time-dependent ROC analysis and C-index were performed to compare the nomogram with CONUT and PNI scores in the training and validation groups.

**Figure 2:**
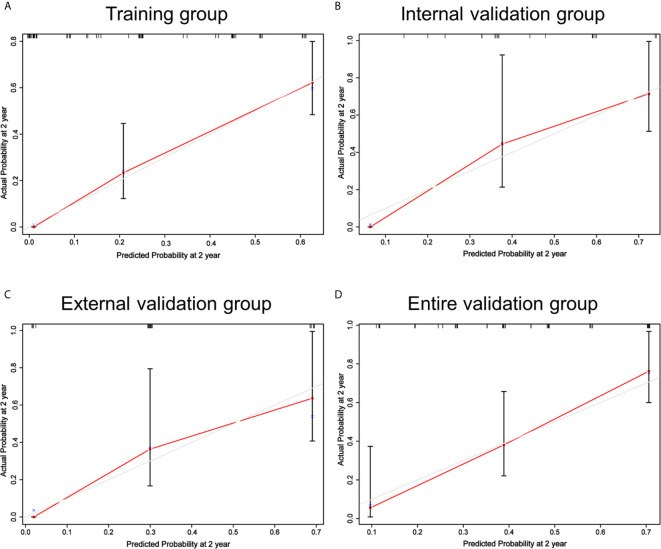
Calibrations of the nomogram for 2-year OS in the training **(A)**, internal validation **(B)**, external validation **(C)** and entire validation groups **(D)**. The tint line at 45° represent perfect prediction and the red line represent actual prediction of our nomogram.

The nomogram was in good agreement between predicted probability and actual probability in the training and validation groups (**Figure 2**). Through R language, the C-index for the nomogram was calculated to be 0.777 in the training group, 0.769 in the internal validation group, 0.759 in the external validation group and 0.774 in the entire validation group, which were all higher than CONUT and PNI scores in the same groups, respectively (**Table S1**).

**Figure 3:**
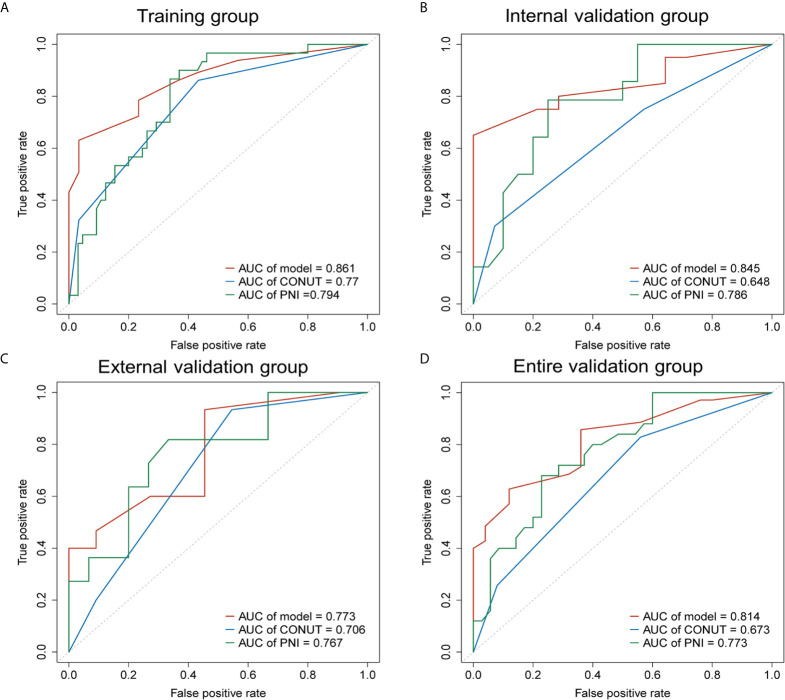
Receiver operating characteristic curves (AUCs) of the nomogram, CONUT and PNI for predicting 2-year OS of patients with resected PDAC in the training, internal validation, external validation and entire validation groups. **(A)** AUCs in the training group; **(B)** AUCs in the internal validation group; **(C)** AUCs in the external validation group; **(D)** AUCs in the entire validation group.

The AUC of the nomogram was 0.861 in the training group, 0.845 in the internal validation group, 0.773 in the external validation group and 0.814 in the entire validation group, respectively, all of which were higher than CONUT and PNI scores of the corresponding groups (**Figure 3**). In the training group, the sensitivity of the nomogram and PNI were both 63.077%, *versus* 86.154% in CONUT, while the specificity of the nomogram was higher than both CONUT and PNI. In the internal validation group, the specificity of the nomogram and CONUT were both 65.000%, *versus* 75.000% in PNI, while the sensitivity of our model was higher than both CONUT and PNI. In the external validation group, the specificity of the nomogram and CONUT were 54.545%, *versus* 81.818% in PNI, while the sensitivity of our model was 93.333% and not lower than CONUT and PNI. In the entire validation group, the sensitivity of the nomogram was 62.857% *versus* 68.571% and 77.143% in CONUT and PNI, respectively, while the specificity of the nomogram was 88.000% and higher than both CONUT and PNI (**Table 3**).

**Table 3:** Diagnostic performance of different models for predicting the prognosis of pancreatic ductal adenocarcinoma.

	AUC (95%CI)	Sensitivity [%]	Specificity [%]	PPV [%]	NPV [%]	Accuracy [%]
***Training group***						
**CONUT**	76.974 (67.975–85.974)	86.154	56.667	81.159	65.385	76.842
**PNI**	79.513 (70.417–88.609)	63.077	90.000	93.182	52.941	71.579
**Model**	86.128 (78.951–93.306)	63.077	96.667	97.619	54.717	73.684
***Internal validation***						
**CONUT**	68.929 (51.342–86.515)	64.286	65.000	56.250	72.222	64.706
**PNI**	78.571 (63.049–94.094)	78.571	75.000	68.750	83.333	76.471
**Model**	84.464 (71.288–97.641)	100.000	65.000	66.667	100.000	79.412
***External validation***						
**CONUT**	70.303 (47.792–92.814)	93.333	54.545	73.684	85.714	76.923
**PNI**	76.667 (57.683–95.651)	66.667	81.818	83.333	64.286	73.077
**Model**	77.273 (58.861–95.684)	93.333	54.545	73.684	85.714	76.923
***Entire validation***						
**CONUT**	70.571 (57.212–83.931)	68.571	64.000	72.727	59.259	66.667
**PNI**	77.314 (65.576–89.053)	77.143	68.000	77.143	68.000	73.333
**Model**	81.429 (70.879–91.978)	62.857	88.000	88.000	62.857	73.333

AUC, area under curve; CI, confidence interval; PPV, positive predictive value; NPV, negative predictive value; CONUT, controlling nutritional status; PNI, prognostic nutritional index.

### NRI and IDI in the Training and Validation Groups

NRI and IDI were established to evaluate the improvement of risk prediction (**Figure S3** and **Table 4**). In the training group, NRI of the nomogram was 0.40 and 0.31 when compared with CONUT and PNI scores respectively, indicating that the predictive ability of the nomogram was 40 and 31% better than CONUT and PNI. Consistently, when compared with CONUT and PNI scores, IDI of the nomogram in the training group was 0.113 and 0.097, revealing the promotion of accuracy by the nomogram in prediction of 2-year survival.

**Table 4:** Diagnostic performance of NRI and IDI between model and CONUT/PNI.

	Continuous NRI (95%CI)		IDI (95%CI)
**Training group**			
model *vs.* CONUT	0.400 (−0.094–0.648)		0.113 (0.025–0.241)
model *vs.* PNI	0.310 (0.018–0.554)		0.097 (0.006–0.221)
**Internal validation**			
model *vs.* CONUT	0.536 (0.036–0.815)		0.271 (0.058–0.436)
model *vs.* PNI	0.343 (−0.087–0.260)		0.079 (−0.087–0.260)
**External validation**			
model *vs.* CONUT	0.394 (−0.313–0.646)		0.094 (−0.077–0.227)
model *vs.* PNI	−0.012 (−0.48–0.534)		0.058 (−0.103–0.563)
**Entire validation**			
model *vs.* CONUT	0.297 (−0.066–0.613)		0.176 (0.023–0.300)
model *vs.* PNI	0.149 (−0.235–0.518)		0.054 (−0.079–0.190)

NRI, net reclassification index; IDI, integrated discrimination improvement; CI, confidence interval; CONUT, controlling nutritional status; PNI, prognostic nutritional index.

Compared with CONUT and PNI, the addition of CA19-9 and diabetes in our nomogram significantly increased the relative NRI and IDI in the training and internal validation groups. Although NRI of the nomogram *versus* PNI in the external validation group was −0.012 which might be due to the limitation of sample size, NRI and IDI of the nomogram were better than both those of CONUT and PNI in the entire validation group.

### Clinical Utility

**Figure 4:**
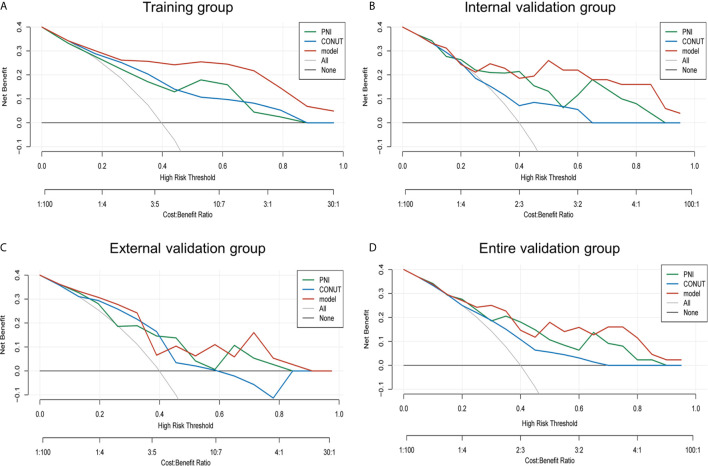
Net benefit curves for predicting 2-year OS based on nomogram as compared with PNI and CONUT model in the training, internal validation, external validation and entire validation groups. **(A)** Net benefit curves based on nomogram as compared with PNI and CONUT model in the training group; **(B)** Net benefit curves based on nomogram as compared with PNI and CONUT model in the internal validation group; **(C)** Net benefit curves based on nomogram as compared with PNI and CONUT model in the external validation group; **(D)** Net benefit curves based on nomogram as compared with PNI and CONUT model in the entire validation group.

The decision curve analysis for the prognosis-related nomogram and that for CONUT and PNI was performed (**Figure 4**). As presented by decision curve in the training group, if the high-risk threshold is >0.1, more benefit could be added than either treat-none-patients scheme or treat-all-patients scheme when using CONUT, PNI or the nomogram, among which the nomogram could achieve the most benefit. Although the nomogram had several overlaps with CONUT and PNI in the internal, external and entire validation groups, it was shown that the nomogram had more benefit.

Conceivably, the nomogram model had better clinical utility than CONUT and PNI scores in 2-year OS.

### Prognostic Stratification Based on the Nomogram for PDAC

**Figure 5:**
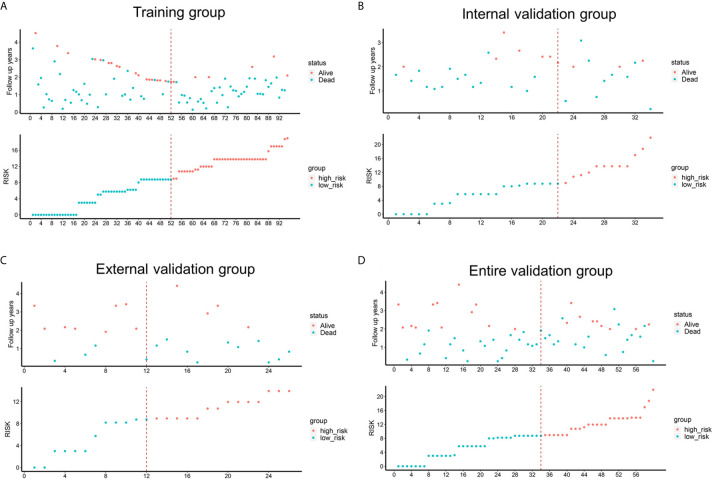
Risk factor correlation diagrams of the training, external validation, internal validation and entire validation groups. The red dots in the figure represented the surviving PDAC patients, and the blue dots represented the dead PDAC patients. The dotted line represented the median value of risk score. The left side of the dotted line represented the low-risk group, and the right side of the dotted line represented the high-risk group. **(A)** risk factor correlation diagram of the training group; **(B)** risk factor correlation diagram of the external validation group; **(C)** risk factor correlation diagram of the internal validation group; **(D)** risk factor correlation diagram of the entire validation group.

**Figure 6:**
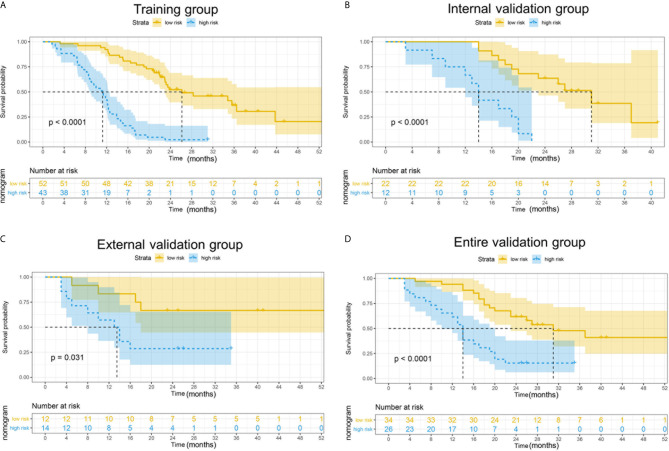
Kaplan–Meier curves for 2-year OS in the training, external validation, internal validation and entire validation groups. **(A)** The survival curves in the training group according to nomogram. The OS probability was significantly worse in the high-risk group compared to the low-risk group (p < 0.0001). **(B)** The survival curves in the external validation group according to nomogram. The OS probability was significantly worse in the high-risk group compared to the low-risk group (p = 0.031). **(C)** The survival curves in the internal validation group according to nomogram. The OS probability was significantly worse in the high-risk group compared to the low-risk group (p < 0.0001). **(D)** The survival curves in the entire validation group according to nomogram. The OS probability was significantly worse in the high-risk group compared to the low-risk group (p < 0.0001).

To determine the prognostic stratification classification for PDAC, the best cut-off value (8.85) for 2-year OS calculated through ROC curve divides patients with resected PDAC into high-risk and low-risk groups (**Figure 5**). Risk factor correlation diagram showed that the patients in high-risk group have poorer survival and higher risk of death. The median survival time of high-risk and low-risk groups were 11 and 26 months in the training group, 14 and 34 months in the internal validation group, 13 and 25 months in the external validation group, 14 and 31 months in the entire validation group, respectively, indicating that the high-risk group displayed a higher frequency of poor survival outcomes than the low-risk group in the training group (*p <*0.0001), internal validation group (*p <*0.0001), external validation group (*p* = 0.031) and entire validation group (*p <*0.0001) (**Figure 6**).

## Discussion

Currently, PDAC is one of the deadliest malignancies with a poor prognosis, to which surgical resection is the sole curable method. On account of this, a specific and convenient approach to predict outcomes and assist to guide doctors when and how to treat patients with PDAC is needed. In the present study, we examined nutritional indicators in PDAC and confirmed that albumin and lymphocyte count were independent protective nutritional indicators while CA19-9 and diabetes were independent risk factors for PDAC. We established a convenient and specific nomogram to predict OS of patients with PDAC and facilitate medical decision-making using the four independent prognostic indicators above. The nomogram showed great abilities of calibration and discrimination in both training and validation groups. Furthermore, our nomogram showed improved prognostic reliability, accuracy and better net benefit when compared with CONUT and PNI scores.

It was thought that cancer prognosis was associated with not only tumor markers and cancer stage, but also preoperative indicators, including inflammation and nutritional status (5). Malnutrition is a serious problem occurring often in patients with PDAC (e.g., due to gastrointestinal obstruction, biliary obstruction, chemotherapy, anorexia). A prospective study reported that 33% of 1,000 patients with cancer were at high nutrition risk, especially in those with pancreatic cancer (13). Numerous studies also have proved that pancreatic cancer patients with poorer preoperative nutritional status have more perioperative complications and poorer long-term survival (14). Therefore, early detection of nutrition status is critical to predicting of prognosis of patients with pancreatic cancer and assisting clinical decision in treatment options. Recent studies indicated that CONUT and PNI scores could be used to predict the long-term survival of patients with PDAC (15–17). The prognostic significance of CONUT in patients with PDAC after surgical resection was identified by Kato et al. (7). Kanda et al. (18) reported that PNI could be used to predict outcomes of patients with PDAC. However, CONUT and PNI were not originally developed for patients with PDAC and may ignore PDAC-specific indicators when assessing prognosis of patients. Therefore, we constructed a novel model to specifically predict OS of patients with PDAC and facilitate clinical decision.

Albumin, as a commonly used indicator for nutritional status, was incorporated in our nomogram. A retrospective study revealed that albumin was a potential indicator to predict the prognosis of patients with resected PDAC (19). Similarly, another retrospective study of 53 patients with pancreatic cancer indicated that patients with preoperative albumin lower than 2.8 g/dl (*p* = 0.021) had a poor prognosis (20). Lymphocyte count has been widely used as an indicator for nutritional and immune status. A cross-sectional study revealed that lymphocyte count could act as a nutritional marker (21). In addition, a multicenter study identified that low preoperative lymphocyte count was an independent prognostic factor for OS of patients with resected pancreatic cancer (22). Previous studies have identified that CA19-9 is a reliable and validated indicator for predicting prognosis of patients with pancreatic cancer (23, 24). Also, Wang et al. (25) proved that serum CA19-9 levels may act as an independent factor to predict postoperative survival of patients with PDAC. Therefore, CA19-9 which is included in our model could increase specificity and sensitivity of the nomogram. Diabetes was another risk factor listed in our model. Accordingly, diabetes had 40% prevalence in patients with pancreatic cancer and was often new on-set (26, 27). Another review indicated that diabetes was risk factor for PDAC development and progression (28).

In the present study, through multivariate Cox regression and LASSO analysis, we identified that albumin, lymphocyte count, CA19-9 and diabetes might be independent prognostic indicators for predicting OS of patients with resected PDAC. By incorporating these four indicators, a novel nomogram to predict 2-year survival of patients with resected PDAC was well constructed. Calibration curves and C-index indicated that the calibrations and discriminations of our nomogram for OS were better than both PNI and CONUT scores in the training and validation groups. Besides, our data showed that the discriminations were excellent in the training, internal validation, entire validation groups and acceptable in the external validation group. Since CONUT and PNI were classic and widely used nutrition indices, we compared our nomogram with them. AUC showed that our nomogram was better than CONUT and PNI. NRI is a widely used metric applied to assess the relative ability of two risk models to distinguish between low- and high-risk individuals (29). IDI is commonly applied to compare two risk prediction models and it summarizes the extent a new model increases risk in events and decreases risk in non‐events (30). NRI and IDI could highlight better the added risk predictive ability of biomarkers compared with established models (31). Besides, a study reported that NRI and IDI have been proposed as alternatives to the increase in the AUC for evaluating improvement in risk assessment (32). In our study, NRI in the training, internal validation and entire validation group showed that the predictive accuracy of our model was higher than PNI and CONUT scores though NRI of our model *versus* PNI in the external validation group was −0.012, which might be due to small sample size of external validation group. Additionally, IDI indicated that the novel constructed nomogram improved accuracy compared with PNI and CONUT scores. The decision curve analysis showed that patients will receive more net benefit from application of the nomogram than CONUT and PNI in the training group (within the threshold range of 0.3–1.0), internal validation group (within the threshold range of 0.5–1.0), external validation group (within the threshold range of 0.5–0.6 and 0.7–0.8), and entire validation group (within the threshold range of 0.5–0.8). Moreover, the model could distinguish patients into low-risk (score <8.85) and high-risk (score >8.85) groups according to the best cut-off point (8.85). Patients with higher scores had shorter survival time than those with lower scores. The cut-off point can facilitate surgeons in decision making to perform radical therapy or other treatments. Chemotherapy, immunotherapy or other treatments may be suitable for patients in the high-risk group, while patients in the low-risk group could take radical therapy and further treatments into consideration. More importantly, the convenient and specific nomogram may have significant clinical usefulness in medical decisions including treatment options, nutrition support and postoperative surveillance.

Although the established and validated nomogram in our study might perform as a convenient and specific prediction tool to assist doctors in clinical decisions, there are some limitations to be acknowledged. First, as a retrospective study, selection and detection bias might exist. Second, it is impossible to standardize postoperative chemotherapy for all patients with PDAC, and the patients included in this study were stages I–III patients with radical surgery, thus the nomogram might not be suitable for all pancreatic ductal adenocarcinoma cases.

## Conclusion

In conclusion, we established and validated a nomogram based on preoperative prognostic indicators to estimate nutritional status and predict OS in patients with resected PDAC, and proved that the nomogram might improve predictive ability, sensitivity and accuracy compared with PNI and CONUT scores. As a convenient and specific tool, it might be of great significance of clinical utility in guiding clinical decisions.

## Data Availability Statement

The raw data supporting the conclusions of this article will be made available by the authors, without undue reservation.

## Ethics Statement

The studies involving human participants were reviewed and approved by the Ethics Committee of the First Affiliated Hospital of Soochow University. The patients/participants provided their written informed consent to participate in this study.

## Author Contributions

Conceptualization: JZ, HL, FZ, and ZC. Methodology: JZ, HL, and FZ. Investigation: FZ, ZC, YT, YH, and YL. Data curation: BY and JY. Writing—original draft preparation: HL. Writing—review & editing: JZ, PD, and DZ. Supervision: JZ. Funding acquisition: JZ and JY. All authors contributed to the article and approved the submitted version.

## Funding

This study was supported by the Project of National Natural Science Foundation of China (grant number: 81902385), the Foundation Research Project of the Natural Science Foundation of Jiangsu Province (grant number: BK20201173), the Jiangsu Provincial Medical Youth Talent (grant number: QNRC2016734), and the Project of Medical Research of Jiangsu Province (grant number: Y2018094).

## Conflict of Interest

The authors declare that the research was conducted in the absence of any commercial or financial relationships that could be construed as a potential conflict of interest.
