# Supplementary material for: Development and Validation of a Nomogram Based on Nutritional Indicators and Tumor Markers for Prognosis Prediction of Pancreatic Ductal Adenocarcinoma
Source: Front Oncol. 2021 May 31;11:682969. doi: 10.3389/fonc.2021.682969 (PMC8200845; doi:10.3389/fonc.2021.682969)
Supplement: Supplementary file 1 [file DataSheet_1.docx]

Supplementary Material

**Supplementary Figures**


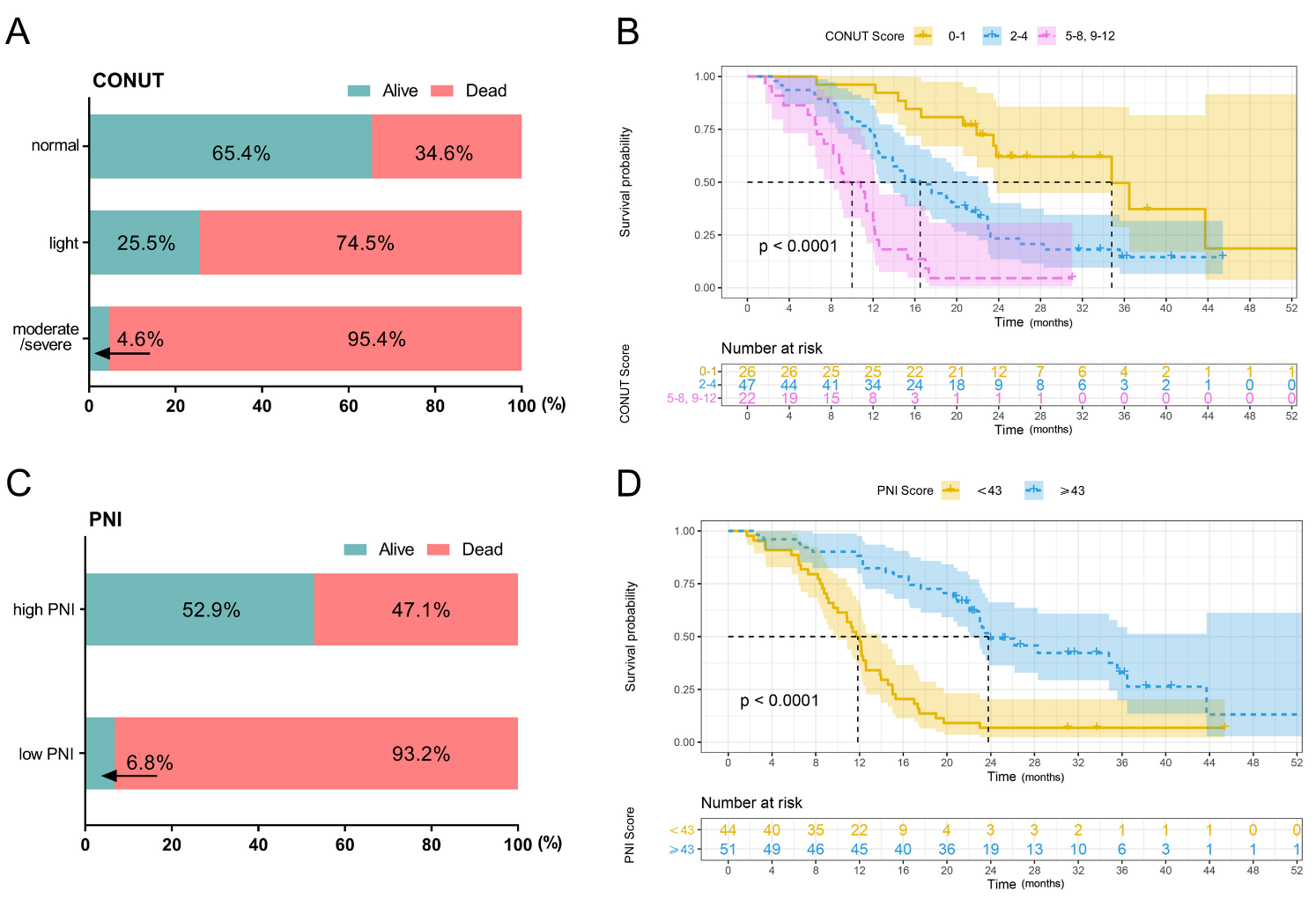


**Supplementary Figure 1.** Survival rates and Kaplan Meier curves of patients in different groups according to CONUT and PNI scores. **(A)**. Survival rates of patients in three CONUT groups; **(B)**. Survival curves of patients in normal, light, moderate and severe groups; **(C)**. Survival rates of patients in high and low PNI groups; **(D)**. Survival curves of patients in low PNI group and high PNI group.


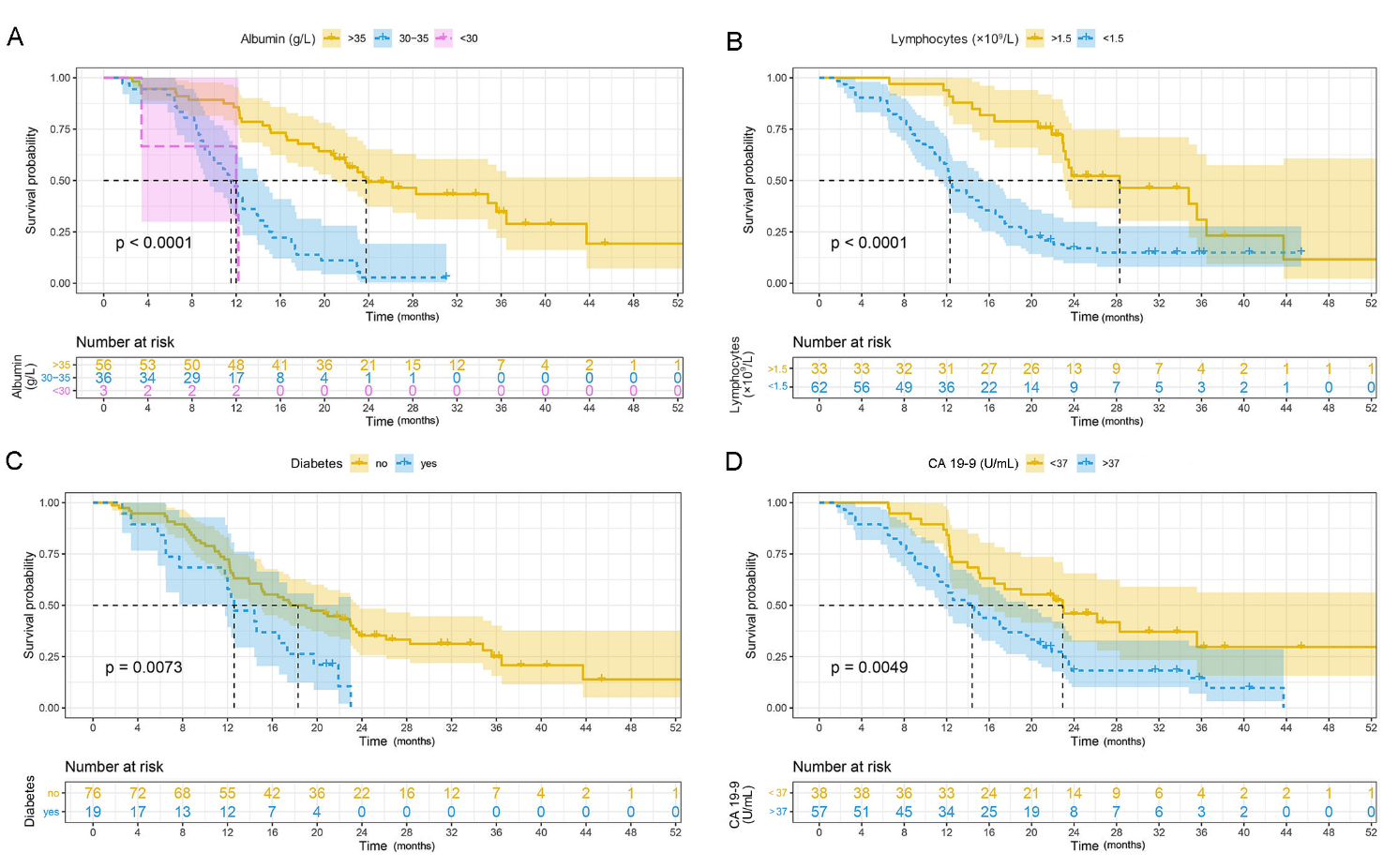


**Supplementary Figure 2.** Kaplan-Meier curves for 2-year overall survival in the training group. **(A)**. The survival curves according to serum albumin level. The overall survival probability was significantly worse in the low serum albumin level group with albumin ≤30U/mL and the medium serum albumin level group with albumin range from 30-35U/mL compared to the high serum albumin level group with albumin ≥35U/mL (*p*<0.0001); **(B)**. The survival curves according to lymphocyte count. The overall survival probability was significantly worse in the low lymphocyte count group compared to the high lymphocyte count group (*p*<0.0001); **(C)**. The survival curves according to diabetes. The overall survival probability was better in the group without diabetes compared to the group with diabetes (*p*=0.0073); **(D)**. The survival curves according to CA19-9 level. The overall survival probability was significantly worse in the high CA19-9 level group compared to the low CA19-9 level group (*p*=0.0049).


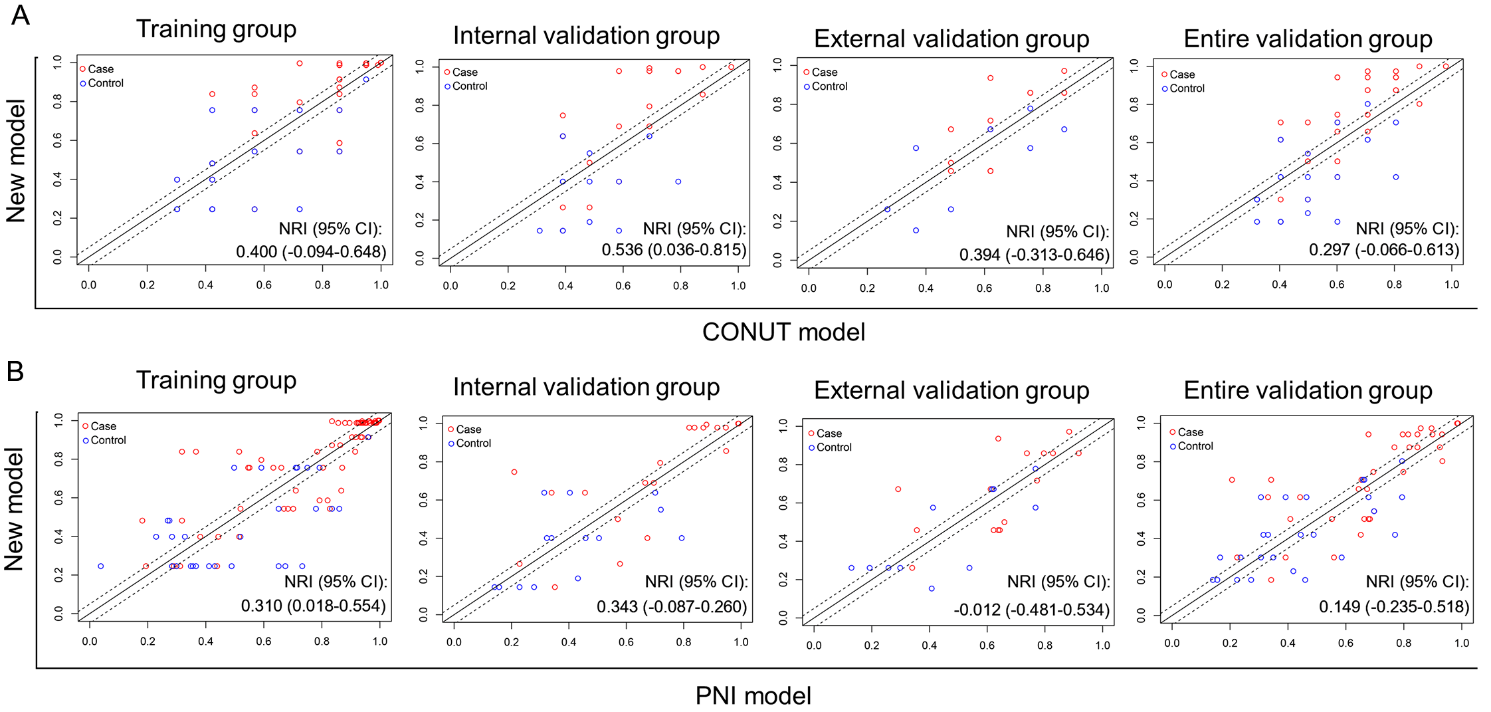


**Supplementary Figure 3.** Net Reclassification Index of the nomogram versus CONUT and PNI model. **(A)**. Net Reclassification index of the nomogram vs. CONUT model; **(B)**. Net Reclassification index of the nomogram versus PNI model. *Abbreviations: NRI, net reclassification index; CI, confidence interval.*

**Supplementary Table**

**Table S1.** Harrell’s concordance indexes of the nomogram, CONUT and PNI in the training and validation groups

|  | Nomogram | CONUT | PNI |
| --- | --- | --- | --- |
| **Training group（n=95）** | 0.777 | 0.719 | 0.731 |
| **Internal group（n=34）** | 0.769 | 0.629 | 0.704 |
| **External group（n=26）** | 0.759 | 0.705 | 0.695 |
| **Entire group（n=60）** | 0.774 | 0.639 | 0.694 |
